# Effect of Lumbar Epidural Steroid Injections on Osteoporotic Fracture and Bone Mineral Density in Elderly Women with Diabetes Mellitus

**DOI:** 10.1155/2020/1538029

**Published:** 2020-12-05

**Authors:** Minsoo Kim, Jiwon Bak, Sejin Kim, Hee-Jeong Son, Seong-Sik Kang, Jin Hue, Byeongmun Hwang, Seung Koo Lee

**Affiliations:** ^1^Department of Anesthesiology and Pain Medicine, Kangwon National University Hospital, School of Medicine, Kangwon National University, Chuncheon 24341, Republic of Korea; ^2^Department of Anatomic Pathology, School of Medicine, Kangwon National University, Kangwon National University Hospital, Chuncheon 24341, Republic of Korea

## Abstract

The incidence of osteoporosis and diabetes mellitus (DM) is known to increase with aging. DM is associated with osteoporotic fractures and decreased bone mineral metabolism. However, no studies have compared the effects of DM on the changes in bone mineral density (BMD) and osteoporotic fracture after epidural steroid injections (ESIs). The present study aimed to analyze the relationship between ESI and BMD changes in elderly women with and without DM. The medical records of elderly women who underwent ESI were retrospectively analyzed. All patients had radiographic and BMD assessments performed before and after receiving lumbar ESIs. A total of 172 patients were divided into two groups according to the presence of DM. The duration of BMD monitoring was 16.1 and 16.8 months in the non-DM and DM groups, respectively. The mean total number of ESIs was 3.4 and 3.2, and the mean cumulative administered dose of glucocorticoids (dexamethasone) was 17 and 16 mg in the non-DM and DM groups, respectively. There were no significant differences between baseline and posttreatment BMD in the lumbar spine, total femur, and femoral neck region in either group. The incidence of osteoporotic fractures at the hip joint and thoracolumbar spine was not significantly different in both groups. ESIs could be used without concerns regarding osteoporosis and fractures in elderly women with DM if low doses of glucocorticoids are used.

## 1. Introduction

Treatment that involves glucocorticoids can cause bone loss and osteoporotic fractures [1]. As the use of epidural steroid injections (ESIs) increases, the side effects of steroid use associated with bone loss are becoming an important issue among pain physicians [2]. Previous studies have reported the effects of ESIs on bone mineral density (BMD) and osteoporotic fractures [3–5], finding that high cumulative doses of glucocorticoid seem to cause a decrease in BMD [3–5]. Osteoporosis and osteoporotic fractures are common problems, especially among elderly women [6, 7]. Therefore, the use of ESIs in elderly women is an important issue with regard to the risk of bone loss and osteoporotic fractures.

The global population is rapidly aging. The incidence of osteoporosis and diabetes mellitus (DM) is known to increase with aging. DM is associated with osteoporotic fractures and decreased bone mineral metabolism [8]. Furthermore, fractures are independently associated with mortality in patients with DM [9]. Therefore, ESIs should only be administered to elderly women with DM after careful consideration. While it may be preferable to avoid glucocorticoids entirely in patients with DM, the nonglucocorticoid treatments are less effective in patients with back pain. Therefore, glucocorticoids are often used in clinical situations [2]. Several studies have reported that low doses and few ESIs are not associated with decreased bone density and fractures [3, 5]. However, even with these more reserved treatments, patients with DM may still have various sequelae. Furthermore, the effects of DM on osteoporotic fracture and BMD in elderly women receiving ESIs have not been adequately studied.

In the present study, we aimed to explore whether DM was associated with BMD and osteoporotic fractures in elderly women who received ESIs for low back pain.

## 2. Methods

### 2.1. Study Population

The present study is a retrospective analysis of elderly women who received ESI for low back pain at the pain management practice center of the Kangwon National University Hospital (Gangwon-do, South Korea) between July 2009 and June 2019. The study was designed according to the STROBE (Strengthening the Reporting of Observational Studies in Epidemiology) guidelines [10] and was approved by the local institutional review board (KNUH-A-2019-12-010-001).

The inclusion criteria for the study were postmenopausal women of ≥50 years who had received ESI for low back pain or lumbar radiculopathy symptoms lasting at least 3 months and who had radiography and BMD assessments performed before and after receiving ESI. The exclusion criteria were a history of comorbidities, such as cancer, pituitary diseases, thyroid disease, rheumatic disease, renal failure, or adrenal disease; previous osteoporotic fracture; fractures due to known accidental traumas; type-1 DM; uncontrolled diabetes with complications; and lumbar spine or femoral surgery, known to affect bone metabolism. Diabetes was defined based on the American Diabetes Association criteria [11]. We included in the present study patients with a diagnosis of type-2 DM on active therapy and with available hemoglobin A1c levels obtained within 3 months before enrollment.

**Figure 1:**
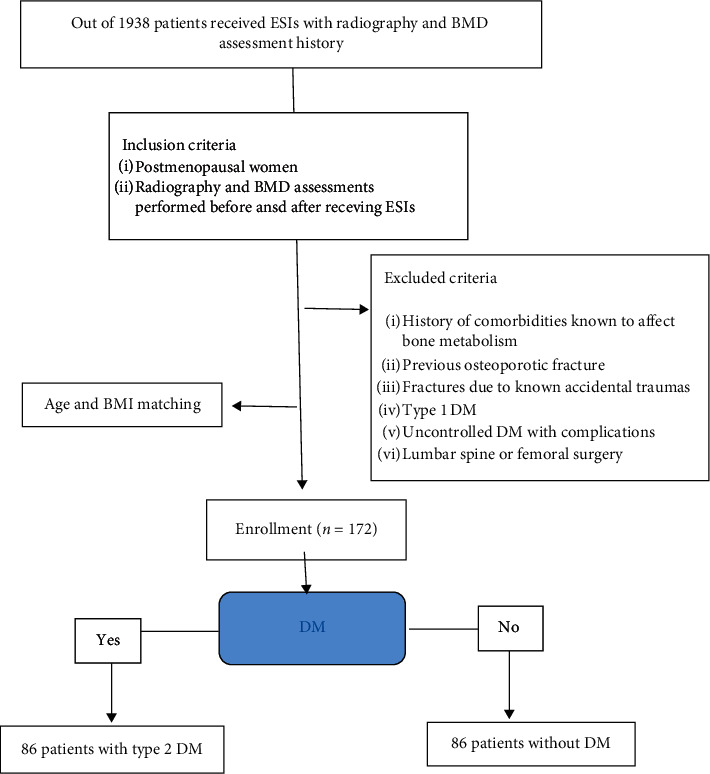
CONSORT flow diagram.

Out of 1938 patients that received ESIs and had radiography and BMD assessments available, 172 patients were selected as participants who satisfied the inclusion criteria after age (±1 year) and body mass index (BMI) matching (Figure 1). The study participants were divided into two groups of 86 participants each: a DM group and a non-DM group. The medications received by patients with DM included metformin, vildagliptin, gliclazide, dapagliflozin, and supplementary drugs (atorvastatin, rosuvastatin, and lorcaserin). The treatments administered for osteoporosis included bisphosphonates, calcium supplements, and hormone replacement therapy. The BMD values of patients with DM were retrieved from periodical assessments conducted during visits to the endocrinology clinic. A questionnaire was administered to each participant to collect information on age at menopause, medical history, smoking and drinking status, and physical activity and exercise habits. Assessment of physical activity was performed as previously described [3]. The participants' occupation was not considered as most of them were housewives and did not hold other jobs. The Verbal Numeric Pain Rating Scale (VNRS) was utilized to compare the changes in pain intensity before ESI and 4 weeks after the last ESI. VNRS measured the pain experienced with 0 representing no pain and 10 representing the worst pain.

The ESIs and the BMD measurements were performed as previously described [3, 12]. In brief, the ESIs, consisting of a mixture of 8 ml lidocaine hydrochloride (0.5%, preservative-free) and dexamethasone, were administered at the lumbar spine level. All participants initially received 1–3 ESIs at 2-week intervals. Additional lumbar ESIs were administered based on the participant's response to prior injections. The lumbar spine (L1–L4), femoral neck, and total femur were the points to which you referred to measure BMD. The BMD was measured by dual-energy X-ray absorptiometry using a Lunar Prodigy system (GE Healthcare, Chicago, IL) and expressed as absolute values (g/cm^2^) and T-scores. The measurements were made before and after treatment. To control this intergroup difference, the change in BMD for each patient was annualized.

### 2.2. Sample Size

The sample size was determined based on previous assessments and studies [12]. The primary outcome of the study was the change in lumbar, femoral neck, and total femur BMD from baseline after ESI in patients with and without DM. Considering a 0.05 two-sided significance level, a power of 90%, an allocation ratio of 1 : 1, and an effect size of 0.5, 86 patients in each group were estimated to be required.

### 2.3. Statistical Analysis

The data were presented as the mean ± standard deviation. Comparisons of the means of demographic and clinical data between the two groups were made using Student's *t*-test. Within each group, changes in BMD compared to baseline were analyzed by a paired *t*-test. The chi-square test and Fisher's exact test were used to compare the differences in categorical variables between the two groups. In all the comparisons, a *P* value <0.05 was considered statistically significant. Statistical analyses were performed using SPSS 25.0 (IBM Corporation, Armonk, NY).

## 3. Results

A total of 172 patients were enrolled in the present study. The mean age was 70.1 ± 7.0 years and 70.0 ± 6.9 years in the non-DM and DM groups, respectively. The patients' baseline characteristics are presented in Table 1. There were no significant differences in age, weight, and BMI between the two groups (Student's *t*-test). Similarly, the baseline BMD of the lumbar spine, total femur, and femoral neck was similar in both the groups. The mean total number of ESIs was 3.4 ± 1.2 and 3.2 ± 1.1, and the mean cumulative administered dose of corticosteroids (dexamethasone) was 17 ± 3.2 and 16 ± 3.4 mg in the non-DM and DM groups, respectively. Duration of BMD monitoring was 16.1 ± 6.2 and 16.8 ± 6.4 months in the non-DM and DM groups, respectively. Mean value of the VNRS decreased to 2.2 ± 2.0 and 2.1 ± 1.8 in the non-DM and DM groups, respectively. The average duration of morbidity in patients with DM was 5.6 ± 5. 3 years, and the mean glycosylated hemoglobin (HbA1c) level was 7.7% ± 1.2 (normal range, ∼5.6%).

The lifestyle characteristics of the participants (smoking, alcohol consumption, exercise, and physical activity) also showed no statistically significant differences between the groups (Table 2) (chi-square test and Fisher's exact test).

**Figure 2:**
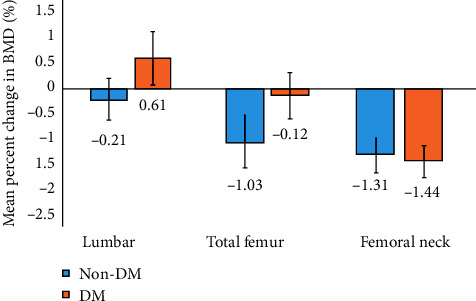
Mean percent change in bone mineral density from baseline after epidural steroid injections. Values are presented as mean ± SD. There were no significant differences between baseline and posttreatment BMD values. The values below bar graphs represent the mean percent change in BMD. The non-DM group consisted of postmenopausal women without diabetes mellitus who received ESI. The DM group consisted of postmenopausal women with diabetes mellitus who received ESI. Bone mineral density data are based on *T*-scores. BMD = bone mineral density; ESI = epidural steroid injection.

The mean change in BMD after ESI in the non-DM and DM groups was −0.21% ± 0.42 and 0.61% ± 0.53 in the lumbar spine, −1.03% ± 0.49 and −0.12% ± 0.45 in the total femur, and −1.31% ± 0.35 and −1.44% ± 0.31 in the femoral neck region, respectively (Figure 2). There were no significant differences between baseline and posttreatment BMD values (paired *t*-test).

The BMD outcomes (normal, osteopenia, or osteoporosis) in the lumbar spine, total femur, and femoral neck regions before and after ESI for both groups are listed in Table 3. In the lumbar spine, the prevalence of osteoporosis before and after ESI was 40% and 41% in the non-DM group and 42% and 40% in the DM group, respectively. In the total femur region, the prevalence before and after ESI was 22% and 27% in the non-DM group and 28% and 24% in the DM group, respectively. In the femoral neck, the prevalence of osteoporosis before and after ESI was 26% and 27% in the non-DM group and 29% and 30% in the DM group, respectively. There were no significant differences in follow-up BMD outcomes between the groups (chi-square test).

The prevalence of fractures in elderly women receiving ESIs is presented in Table 4. The incidence of thoracolumbar spine and hip joint fractures in the non-DM and DM groups was 6% and 5% and 1% and 1%, respectively. There were no significant differences in the prevalence of osteoporotic fracture between the groups (Fisher's exact test).

## 4. Discussion

The number of patients with DM and patients receiving ESIs is increasing with the increase in the elderly population. However, no studies have been conducted on the interactions between DM and ESIs in elderly individuals. The present study analyzed the effects of DM on the change in BMD and the incidence of osteoporotic fractures in elderly women who received ESIs for low back pain. We found that BMD at a mean of 16 months after ESIs in postmenopausal women was not significantly different compared to previous values in both groups (with and without DM). The results show that, in patients matched for age, BMI, and lifestyle, the incidence of osteoporotic fractures at the hip joint and thoracolumbar spine did not significantly differ according to the presence or absence of DM.

Recent data seem to suggest that DM can be a determinant of bone health [13]. Previous studies have suggested that various diabetes-related factors are associated with the risk of bone fragility subsequent to impaired bone formation and bone remodeling [14, 15]. Patients with type-2 DM could experience fragility fractures even without a loss of BMD because of the deterioration of bone quality [8, 9, 13, 14]. DM also leads to an increased risk of fracture, particularly in women [8, 9]. The use of steroids in patients with diabetes is controversial. Among Korean pain physicians, more than half reported that they used fewer corticosteroids (an average of 0.6 fold of the total steroid dose used in patients without diabetes) for ESIs in patients with diabetes [1]. At low cumulative doses of glucocorticoids, several studies reported no significant relationship between ESI, BMD, and vertebral fractures [5, 12, 16]. In contrast, Kim and Hwang found that frequent ESIs (a mean number of 14 and a mean total dose of triamcinolone 400 mg) might reduce BMD in elderly women [3]. Glucocorticoid-induced bone loss is reported to be associated with the duration and dose of glucocorticoid administration [2, 17–19]. In the present study, the patients received ESIs at a low frequency (mean number of 3.4 and 3.2) with a low total dose (mean total dose of dexamethasone, 17 and 16 mg). The change in BMD value after ESIs is similar in both the groups. Considering the result of our study, infrequent ESIs with low total doses do not seem to affect BMD in elderly women with type-2 DM.

Change in BMD with glucocorticoid use occurs rapidly in the first 6 months of therapy, followed by a slower decline [17, 18]. Nah et al. found that BMD decreased significantly during the first year in patients receiving ESIs [16]. Therefore, we believe that our study period (mean 16 months) was sufficient for measuring changes in BMD. In a previous study, the mean decrease in BMD in the lumbar spine, femoral neck, and greater trochanter in the placebo group after 1 year was approximately 3% [20]. The change in BMD in Korean postmenopausal women was approximately −1% in the lumbar spine during a 1-year period [21]. Kang et al. showed that the mean change in BMD in postmenopausal women receiving ESIs (mean duration of BMD measurement, 14 months; number of injections, 5.6 times; and mean total dose of triamcinolone, 212 mg) was 0.06% in the lumbar spine, −1.57% in the total femur region, and −2.87% in the femoral neck [22]. In Kim et al.'s study (mean duration of BMD measurement, 19 months; number of injections, 3.6; and mean total dose of dexamethasone, 9 mg), the mean change was 0.69% in the lumbar spine, −2.23% in the total femur region, and −1.48% in the femoral neck [23]. In the present study, the mean change in BMD in patients receiving ESIs (mean duration of BMD measurement, 16 months; number of injections, 3.4-3.2; and mean total dose of dexamethasone, 17-16 mg) was −0.12% and 0.61% in the lumbar spine, −1.03 and −0.12 in the total femur region, and −1.31 and −1.44 in the femoral neck in the non-DM and DM groups, respectively. In the present study, the change in the BMD value is similar to the results of previous studies [21–23]. However, the change in BMD varied from patient to patient. The above data may indicate that the patient's management status has more influence on BMD than do the ESIs. Additionally, contrary to our expectations, patients with DM experienced a less pronounced decrease in BMD in the total femur and lumbar spine region than those without DM. This may be because patients with DM were more actively managed than those without, including care under the supervision of an endocrinologist. Kim et al. reported that the lumbar spine is significantly less affected by ESIs than the femur [23], which is similar to our own findings. They posited that ESI-induced increase in physical activity is the reason for the less change in BMD caused by corticosteroid in the lumbar spine [23].

Cui et al. reported that the prevalence of osteoporosis in postmenopausal Korean women was 51% in the lumbar spine and 11% in the femoral neck [24]. In postmenopausal Korean women receiving ESIs, previous studies had reported that the prevalence of osteoporosis was 28–68% in the lumbar spine, 22–41% in the total femur, and 23–31% in the femoral neck [5, 16, 23]. In the present study, in the patients without and with DM, the prevalence of osteoporosis was 40% and 42% in the lumbar spine, 22% and 28% total femur, and 26% and 29% in the femoral neck, respectively. There was no change in the prevalence of osteoporosis after ESIs. The findings of the present study showed similar results to those of previous studies in terms of the prevalence and anatomical distribution of osteoporosis [5, 16, 24].

Exogenous glucocorticoids, old age, and lower BMD are major risk factors for osteoporotic fractures [25, 26]. The risk of fracture appears within a few months of starting glucocorticoid treatment [16, 17, 23]. Nah et al.'s study of postmenopausal Korean women receiving ESI found that the incidence of new fractures over a 2-year period was 15% in the spine, 1% in the hip, and 10% in the radius [16]. In the present study, in the patients without and with DM, the incidence over 16 months was 6% and 5% in the spine, 1% and 2% in the hip, and 2% and 0% in the ulnar and radius, respectively. Thus, our findings regarding spine and hip joint fractures were similar to those of Nah et al. In light of our results, it can be seen that the use of glucocorticoids at low dose through few ESIs does not have a significant effect on the incidence of osteoporotic fractures and BMD changes in postmenopausal women with DM. However, since glucocorticoid-induced bone loss is duration- and dose-related [2, 17–19], frequent ESIs and the use of high doses may increase the incidence of osteoporotic fractures and decreased BMD.

The presence of DM may affect BMD and the incidence of osteoporotic fracture [13, 14]. The risk of hip fracture in patients with type-2 DM is 1.4–1.7-fold higher than in healthy individuals [27, 28]. Another study found that type-2 DM was associated with an increased risk of vertebral fractures in women [29]. However, Dytfeld and Michalak reported that type-2 DM in postmenopausal women was associated with a higher risk of hip fracture, but not vertebral fracture [30]. Considering the change in BMD observed in the present study, Dytfeld et al.'s results may be explained by the minor decrease in lumbar spine BMD. Since DM may affect bone density and fractures, the most important remedial factor is proper control of blood glucose levels. Poor glycemic control is associated with the risk of osteoporotic fractures [31–33]. Kim et al. reported that postmenopausal women receiving ESIs with antiosteoporotic medication had no changes in BMD [23]. Therefore, antiosteoporotic management and glycemic control should be considered for postmenopausal women with DM receiving ESI.

Wong et al. showed that pain reduction after ESI did not differ significantly according to the presence of DM [34]. Similarly, in a study of patients that received cervical ESIs, the nondiabetic group had a mean reduction in a pain score of 2.4, and the diabetic group had a mean reduction in a pain score of 2.5 [35]. However, uncontrolled DM may be associated with decreased pain reduction after ESI. Furthermore, the resultant reduction in pain may generally decrease with increase in HbA1c levels [32, 34]. In the present study, the patients without DM had a mean reduction in a pain score of 2.2 compared to 2.1 in patients with DM. The mean HbA1c level in patients with DM was 7.7%, and the pain reduction experienced was similar in both groups regardless of DM.

This study had some limitations. First, this study did not include patients that received frequent ESIs with high cumulative doses of glucocorticoids. However, physicians are generally reluctant to recommend long-term ESIs in patients with DM due to fear of adverse events. Second, our study did not report long-term results. Despite the aforementioned limitations, this is valuable as it is the first study to analyze the effects of DM on the change in BMD and the incidence of osteoporotic fractures in elderly women who received ESIs for low back pain.

Based on the results of the present study, we suggest that ESI treatment using low cumulative doses of corticosteroids (less than about 20 mg of dexamethasone) could be used safely, without any significant impact on BMD and osteoporotic fractures, in elderly women with DM.

## 5. Conclusions

In conclusion, we found no statistically significant difference in the incidence of osteoporotic fractures at the thoracolumbar spine and hip joint among elderly women with or without DM who received ESIs for low back pain. In addition, the changes in lumbar spine, femoral neck, and total femur region BMD were also equivalent. ESIs can be administered without concerns regarding osteoporosis and fractures in elderly women with DM if low doses of glucocorticoids are used.

## Figures and Tables

**Table 1:** Baseline demographic and clinical characteristics.

Characteristic	Non-DM group (*n* = 86)	DM group (*n* = 86)	*P*
Age (years)	70.1 ± 7.0	70.0 ± 6.9	0.619

Weight (kg)	54.3 ± 6.1	54.1 ± 6.3	0.525

BMI (kg/m^2^)	23.5 ± 2.7	23.4 ± 2.9	0.421

Change in pain score	−2.2 ± 2.0	−2.1 ± 1.8	0.473

Baseline BMD (g/cm^2^)			
Lumbar spine (L1-L4)	0.900 ± 0.12	0.887 ± 0.13	0.379
Total femur	0.802 ± 0.13	0.785 ± 0.13	0.312
Femoral neck	0.732 ± 0.12	0.698 ± 0.16	0.215

Average number of total ESIs	3.4 ± 1.2	3.2 ± 1.1	0.372

Cumulative glucocorticoid dose (dexamethasone, mg)	17 ± 3.2	16 ± 3.4	0.252

Duration of BMD monitoring (months)	16.1 ± 6.2	16.8 ± 6.4	0.274

Values are presented as mean ± SD. The non-DM group consisted of postmenopausal women without diabetes mellitus who received ESI. The DM group consisted of postmenopausal women with diabetes mellitus who received ESI. Bone mineral density data are based on *T*-scores. BMD = bone mineral density; BMI = body mass index; ESI = epidural steroid injection. There was no significant difference between the two groups.

**Table 2:** Lifestyle characteristics of the patients.

Characteristic	Non-DM group (*n* = 86)	DM group (*n* = 86)
Current smoker	7 (8%)	10 (12%)
Frequent alcohol consumption	14 (16%)	12 (14%)
Regularly exercise	6 (7%)	8 (9%)
Physical activity		
Low	24 (28%)	22 (26%)
Moderate	58 (67%)	61 (71%)
Vigorous	4 (5%)	3 (3%)

Values represent the number of patients (%). The non-DM group consisted of postmenopausal women without diabetes mellitus who received ESI. The DM group consisted of postmenopausal women with diabetes mellitus who received ESI. There was no significant difference between the two groups.

**Table 3:** Prevalence of osteoporosis and osteopenia in postmenopausal women receiving lumbar epidural injections.

	Non-DM group (*n* = 86)		DM group (*n* = 86)	
	Baseline	1-year F/U	Baseline	1-year F/U
Lumbar spine				
Normal	15 (17%)	13 (15%)	12 (14%)	13 (15%)
Osteopenia	37 (43%)	38 (44%)	38 (44%)	39 (45%)
Osteoporosis	34 (40%)	35 (41%)	36 (42%)	34 (40%)

Total femur				
Normal	19 (22%)	19 (22%)	19 (22%)	17 (20%)
Osteopenia	48 (56%)	44 (51%)	43 (50%)	48 (56%)
Osteoporosis	19 (22%)	23 (27%)	24 (28%)	21 (24%)

Femoral neck				
Normal	14 (16%)	13 (15%)	10 (12%)	10 (12%)
Osteopenia	50 (58%)	50 (58%)	51 (59%)	50 (58%)
Osteoporosis	22 (26%)	23 (27%)	25 (29%)	26 (30%)

Values represent the number of patients (%). The non-DM group consisted of postmenopausal women without diabetes mellitus who received ESI. The DM group consisted of postmenopausal women with diabetes mellitus who received ESI. There were no significant differences in follow-up BMD outcomes between the groups. Bone mineral density data are based on *T*-scores. Osteopenia was defined as −2.5 SD < BMD T score < −1.0 SD. Osteoporosis was defined as BMD *T*-score ≤ −2.5 SD. BMD = bone mineral density; ESI = epidural steroid injection; F/U = follow-up.

**Table 4:** Prevalence of fractures in postmenopausal women receiving lumbar epidural injections.

Site of fracture	Non-DM group (*n* = 86)	DM group (*n* = 86)
Thoracolumbar spine	5 (6%)	4 (5%)
Hip joint	1 (1%)	2 (2%)
Ulnar and radius	2 (2%)	0 (0%)
Others	0 (0%)	1 (1%)

Values represent the number of patients (%). The non-DM group consisted of postmenopausal women without diabetes mellitus who received ESI. The DM group consisted of postmenopausal women with diabetes mellitus who received ESI. There were no significant differences in the prevalence of osteoporotic fracture between the groups.

## Data Availability

The data used to support the findings of this study are included within the article.

## References

[1] Compston J. (2018). Glucocorticoid-induced osteoporosis: an update. *Endocrine*.

[2] Kim E. J., Moon J. Y., Park K. S. (2014). Epidural steroid injection in Korean pain physicians: a national survey. *The Korean Journal of Pain*.

[3] Kim S., Hwang B. (2014). Relationship between bone mineral density and the frequent administration of epidural steroid injections in postmenopausal women with low back pain. *Pain Research and Management*.

[4] Mandel S., Schilling J., Peterson E., Rao D. S., Sanders W. (2013). A retrospective analysis of vertebral body fractures following epidural steroid injections. *The Journal of Bone & Joint Surgery*.

[5] Yi Y., Hwang B., Son H., Cheong I. Y. (2012). Low bone mineral density, but not epidural steroid injection, is associated with fracture in postmenopausal women with low back pain. *Pain Physician*.

[6] Cannada L. K., Hill B. W. (2014). Osteoporotic hip and spine fractures: a current review. *Geriatric Orthopaedic Surgery & Rehabilitation*.

[7] Nazrun A. S., Tzar M. N., Mokhtar S. A., Mohamed I. N. (2014). A systematic review of the outcomes of osteoporotic fracture patients after hospital discharge: morbidity, subsequent fractures, and mortality. *Therapeutics and Clinical Risk Management*.

[8] Kanazawa I., Sugimoto T. (2018). Diabetes mellitus-induced bone fragility. *Internal Medicine*.

[9] Miyake H., Kanazawa I., Sugimoto T. (2018). Association of bone mineral density, bone turnover markers, and vertebral fractures with all-cause mortality in type 2 diabetes mellitus. *Calcified Tissue International*.

[10] von Elm E., Altman D. G., Egger M., Pocock S. J., Gøtzsche P. C., Vandenbroucke J. P. (2014). The strengthening the reporting of observational studies in epidemiology (STROBE) statement: guidelines for reporting observational studies. *International Journal of Surgery*.

[11] American Diabetes Association (2016). Standards of medical care in diabetes—2016 abridged for primary care providers. *Clinical Diabetes*.

[12] Kim M., Yang Y. H., Son H. J. (2019). Effect of medications and epidural steroid injections on fractures in postmenopausal women with osteoporosis. *Medicine*.

[13] Safarova S. S. (2019). Alterations of bone metabolism in patients with diabetes mellitus. *International Journal of Endocrinology*.

[14] Kanazawa I., Takeno A., Tanaka K.-i., Yamane Y., Sugimoto T. (2019). Osteoporosis and vertebral fracture are associated with deterioration of activities of daily living and quality of life in patients with type 2 diabetes mellitus. *Journal of Bone and Mineral Metabolism*.

[15] Hygum K., Starup-Linde J., Harsløf T., Vestergaard P., Langdahl B. L. (2017). Mechanisms in endocrinology: diabetes mellitus, a state of low bone turnover - a systematic review and meta-analysis. *European Journal of Endocrinology*.

[16] Nah S. Y., Lee J. H., Lee J. (2018). Effects of epidural steroid injections on bone mineral density and bone turnover markers in patients taking anti-osteoporotic medications. *Pain Physician*.

[17] van Staa T. P., Leufkens H. G. M., Abenhaim L., Zhang B., Cooper C. (2000). Oral corticosteroids and fracture risk: relationship to daily and cumulative doses. *Rheumatology*.

[18] Everdingen A. A., Reesema S., Jacobs J., Bijlsma J. (2003). Low-dose glucocorticoids in early rheumatoid arthritis: discordant effects on bone mineral density and fractures?. *Clinical and Experimental Rheumatology*.

[19] Gaber T.-Z., McGlashan K. A., Love S., Jenner J. R., Crisp A. J. (2002). Bone density in chronic low back pain: a pilot study. *Clinical Rehabilitation*.

[20] Cohen S., Levy R. M., Keller M. (1999). Risedronate therapy prevents corticosteroid-induced bone loss. *Arthritis & Rheumatology*.

[21] Lee E. Y., Kim D., Kim K. M. (2012). Age-related bone mineral density patterns in Koreans (KNHANES IV). *The Journal of Clinical Endocrinology & Metabolism*.

[22] Kang S. S., Hwang B., Son H., Cheong I. Y., Lee S. J., Chung T. Y. (2012). Changes in bone mineral density in postmenopausal women treated with epidural steroid injections for lower back pain. *Pain Physician*.

[23] Kim Y. U., Karm M. H., Cheong Y. (2016). Effect of epidural steroid injection on bone mineral density in postmenopausal women according to antiosteoporotic medication use. *Pain Physician*.

[24] Cui L.-H., Choi J.-S., Shin M.-H. (2008). Prevalence of osteoporosis and reference data for lumbar spine and hip bone mineral density in a Korean population. *Journal of Bone and Mineral Metabolism*.

[25] Maroutti N., Corrado A., Cantatore F. P. (2010). Glucocorticoids induced risk of fractures. *Panminerva Medica*.

[26] Kerezoudis P., Rinaldo L., Alvi M. A. (2018). The effect of epidural steroid injections on bone mineral density and vertebral fracture risk: a systematic review and critical appraisal of current literature. *Pain Medicine*.

[27] Janghorbani M., Van Dam R. M., Willett W. C., Hu F. B. (2007). Systematic review of type 1 and type 2 diabetes mellitus and risk of fracture. *American Journal of Epidemiology*.

[28] Vestergaard P. (2007). Discrepancies in bone mineral density and fracture risk in patients with type 1 and type 2 diabetes-a meta-analysis. *Osteoporosis International*.

[29] Yamamoto M., Yamaguchi T., Yamauchi M., Kaji H., Sugimoto T. (2009). Diabetic patients have an increased risk of vertebral fractures independent of BMD or diabetic complications. *Journal of Bone and Mineral Research*.

[30] Dytfeld J., Michalak M. (2017). Type 2 diabetes and risk of low-energy fractures in postmenopausal women: meta-analysis of observational studies. *Aging Clinical and Experimental Research*.

[31] Oei L., Zillikens M. C., Dehghan A. (2013). High bone mineral density and fracture risk in type 2 diabetes as skeletal complications of inadequate glucose control: the Rotterdam Study. *Diabetes Care*.

[32] Li C.-I., Liu C.-S., Lin W.-Y. (2015). Glycated hemoglobin level and risk of hip fracture in older people with type 2 diabetes: a competing risk analysis of Taiwan diabetes cohort study. *Journal of Bone and Mineral Research*.

[33] Schwartz A. V., Margolis K. L., Sellmeyer D. E. (2012). Intensive glycemic control is not associated with fractures or falls in the accord randomized trial. *Diabetes Care*.

[34] Wong F., Namdari B., Dupler S. (2016). No difference in pain reduction after epidural steroid injections in diabetic versus nondiabetic patients: a retrospective cohort study. *Journal of Anaesthesiology Clinical Pharmacology*.

[35] Ma V., Shakir A. (2013). The impact of type 2 diabetes on numeric pain score reduction following cervical transforaminal epidural steroid injections. *Skeletal Radiology*.

